# Antimicrobial Potential of Extract from a *Pseudomonas aeruginosa* Isolate

**DOI:** 10.1155/2022/4230397

**Published:** 2022-06-29

**Authors:** Francis Kwaku Dzideh Amankwah, Stephen Yao Gbedema, Yaw Duah Boakye, Marcel Tunkumgmen Bayor, Vivian Etsiapah Boamah

**Affiliations:** Department of Pharmaceutics, Faculty of Pharmacy and Pharmaceutical Sciences, Kwame Nkrumah University of Science and Technology (KNUST), Kumasi, Ghana

## Abstract

Microorganisms are one of the main sources of antimicrobial agents and over 50% of antibiotics currently used in hospitals are metabolites from microbes. This study aimed to isolate microorganisms from the Dompoase landfill site, Kwame Nkrumah University Physics Garden, Kosiko River, and Ada Foah seashore of Ghana and screen their metabolites for antimicrobial activity. Forty-eight (48) microorganisms were isolated and their metabolites were screened against *Staphylococcus aureus*, *Enterococcus faecalis*, *Escherichia coli*, *Klebsiella pneumoniae*, *Vibrio cholerae, Salmonella typhi*, *Pseudomonas aeruginosa*, *Streptococcus pyogenes, Proteus mirabilis*, and *Candida albicans* using the agar well diffusion method. Ten (10) of the isolates exhibited antimicrobial activity. Isolate DO5, identified as *P. aeruginosa* isolate, from the Dompoase landfill site was selected for fermentation because it exhibited the highest activity against all the test organisms. DO5 produced optimum antimicrobial activity when fermented for 11 days at 30°C. In the agar diffusion method, the extract of isolate DO5 recorded zones of inhibition ranging between 11.67 ± 0.23 and 21.50 ± 0.71 mm. The MIC and MBC recorded for the DO5 extract ranged from 3.13–25.0 mg/mL and from 6.25–50.0 mg/mL, respectively. Column chromatography analysis yielded eight (8) subfractions from the DO5 extract. IR analysis revealed the presence of functional groups such as alcohols, esters, and hydrocarbons in the fractions. GC-MS analysis identified nine compounds that have been reported to have antimicrobial agents. The DO5 metabolites stand the chance to be developed into potent antibiotics for infection treatment.

## 1. Introduction

Antibiotic resistance is one of the most pressing global health issues today. Antibiotic resistance mechanisms frequently occur as a result of misuse of antibiotics in medicine and agriculture in both industrialized and developing nations, posing a threat to modern medicine by reducing the efficacy of therapeutically relevant antibiotics [1–3]. The overuse of antibiotics has put bacteria under long-term selective pressure, resulting in antibiotic resistance, which means the bacteria will be tough to kill. Antibiotic resistance genes (ARGs) are the key functional elements in antibiotic-resistant bacteria and they can move between microbes via horizontal gene transfer [4, 5].

Antibiotic and antimicrobial resistance (AMR) has resulted in increased morbidity and death as a result of treatment failures, as well as higher health-care expenses [1, 2]. Research has it that AMR is expected to become the major cause of death in the world by 2050, with 10 million fatalities every year. As indicated in the 2014 O'Neill report, which was commissioned by the UK government, AMR is anticipated to have a detrimental impact on the global economy, forecasting a decline in global GDP of 2.5% to 3% (up to $100 trillion) between 2014 and 2050 [1]. Thus, antibiotic resistance has been dubbed “the silent tsunami facing modern medicine” [4].

Without the invention of novel antibiotics, and as resistance develops, we may find ourselves in a situation similar to that which existed prior to the advent of antibiotics, where routine medical procedures become extremely risky as a consequence of failure to prevent or treat infections that are presently simple to handle [1].

The broad-spectrum beta-lactamase (ESBL) enzymes found in *Enterobacteriaceae* have also aggravated the global antibiotic-resistance menace. These ESBL enzymes hydrolyze and inactivate beta-lactam antibiotics such as cephalosporins and also enhance microbial resistance to quinolones, aminoglycosides, and sulfonamides leading to the emergence of multidrug resistance (MDR) [5].

This menace has therefore necessitated the search for more potent metabolites from microorganisms that can be developed into antibiotics as treatment options for resistant bacteria infections [5]. *Streptomyces*, *Bacillus, Cephalosporium,* and *Penicillium* which are known producers of antibiotics are being studied for antibiotics production globally [6, 7]. *Actinomycetes* have also been reported to be producers of many biologically active metabolites which have been developed into drugs [8]. Vancomycin and rifampicin are antibiotics obtained from *Actinomycetes* strains against methicillin-resistant *Staphylococcus aureus,* tuberculosis, and leprosy [9]. Many antibiotics such as aminoglycosides, macrolides, *β* lactams, peptides, polyenes, tetracyclines, and anthracycline were obtained from the genus *Streptomyces* [10, 11]. Whereas, gentamicin, loaiviticin A and B, and tetrocarcin antibiotics were isolated from *Micromonospora* strains [12, 13].

Several antimicrobial compounds have been isolated from terrestrial or soil microorganisms. Between 2006 and 2007 alone, about 1,736 anti-infective, anticancer and anti-inflammatory compounds like pestalone, hypoxysordarin, and equisetin were isolated from marine and aquatic environments [14, 15]. However, the rate of finding new bioactive agents from this same environment for drug development has declined significantly [16]. The aquatic environment which comprises many living organisms including microbes is still considered an unexploited reservoir for novel bioactive compounds [17]. This has therefore necessitated the need to search for antimicrobial agents by focusing on antimicrobe-producing microbes from water bodies and soil samples. Hence, the aim of this study was to isolate, identify, and screen the metabolites of antimicrobe- producing microbes from selected soil and water bodies in Ghana.

## 2. Materials and Methods

### 2.1. Sampling

Thirty-six (36) samples comprising sea water, river water, and soil samples were collected from three different sources in Ghana, namely, Dompoase landfill site, Kumasi, Ashanti region; Physics Garden, Kwame Nkrumah University of Science and Technology, Kumasi, Ashanti region; Kosiko River, Swedru, Central Region; and Ada Foah seashore, Ada, Greater Accra region. These samples were transported on ice to the microbiological laboratory of the Department of Pharmaceutics, Faculty of Pharmacy and Pharmaceutical Sciences, Kwame Nkrumah University of Science and Technology (KNUST), Kumasi, Ghana, and stored in a refrigerator. The soil samples were collected at depths of 2–12 cm with a spatula into sterile plastic bags, while the river and seawater samples were collected into sterile 150 mL bottles [18].

### 2.2. Isolation of Pure Colonies from Samples

(1.0 mL) of the water samples was separately pipetted into 9 mL sterile water making a ten-fold dilution. A 100 *μ*L aliquot of samples was spread on 20 mL solidified nutrient agar plates. For soil samples, 1.0 g of the soil was suspended in 9 mL sterile water and diluted 5 times. One hundred microlitres (100 *μ*L) of the suspension was drawn from all dilutions and spread onto separate 20 mL solidified nutrient agar plates (CM0003, Oxoid UK). All the plates were incubated at 37°C (using Incubator 3032-3033, Hamburg, Germany) for 72 hours. After three days of incubation, the culture plates were observed and microbial colonies surrounded with clear zones of inhibition were fished out. All isolates were separately cultured on a sterile nutrient agar slant and stored in a 30% sterile glycerol broth at −4°C in a frost-free refrigerator.

### 2.3. Test Microorganisms

Typed strains (*Staphylococcus aureus* ATCC 25923, *Enterococcus faecalis* ATCC 29212, *Escherichia coli* ATCC 25922, and *Pseudomonas aeruginosa* ATCC 4853) and clinical strains (*Salmonella typhi, Streptococcus pyogenes, Klebsiella pneumoniae, Proteus mirabilis, Vibrio cholerae,* and *Candida albicans)* were obtained from the Microbiology Laboratory of the Department of Pharmaceutics, Faculty of Pharmacy and Pharmaceutical Sciences, KNUST. The test organisms were prepared by streaking all test bacteria on 20 mL sterile nutrient agar and fungus strain on Sabouraud dextrose agar plates followed by incubation at 37°C for 24 h and 25°C for 72 h, respectively. Well-isolated colonies of each test organism were fished and suspended in 10 mL sterile water in test tubes. The turbidity of the suspensions was adjusted to that of the 0.5 McFarland standard.

### 2.4. Primary Screening of Isolated Microorganisms for Antimicrobial Activity

The microbial isolates were evaluated by screening for the production of antimicrobial metabolites using the agar well diffusion method [19]. All the isolated organisms were cultured in 10 mL nutrient broths for eleven days at 37°C. The growth cultures were centrifuged for 15 minutes at 700 revolutions per minute and their supernatants were evaluated for antimicrobial activity against the test microorganisms whose inoculates were prepared and adjusted to the 0.5 McFarland standard. One hundred microliters (100 *μ*L) of the test microbial suspension were inoculated into sterile nutrient agar plates by the pour plate method. Using a sterile cork-borer, five equidistant wells (10 mm in diameter) were created in each agar plate and filled with 200 *μ*L of the microbial supernatant. The plates were kept for 2 h at room temperature for the diffusion of the bioactive metabolite into the plated agar. Two replicates were done and the plates were incubated for 24 h at 37°C. The diameter of zones of growth inhibition produced was measured and mean values were calculated. The isolate which showed the most promising activity (isolate DO5) was selected for further studies.

### 2.5. Biochemical and Morphological Characterization of Isolate DO5

#### 2.5.1. Macroscopic Characterization

The isolate was grown on nutrient agar plates at 30°C for 24 h and its phenotypic characteristics such as color and motility were determined [20]. DO5 colonies appeared gray.

#### 2.5.2. Microscopic Characterization by Gram Staining

Gram's staining was conducted to determine micromorphological characteristics of isolate DO5 using a DM700 light microscope fitted with a camera [21, 22]. Isolate DO5 was grown on a plate of nutrient agar by streaking and incubating at 37°C for 24 h. A colony was picked to prepare a smear on a microscope slide (Sure friend Medicals Middlesex, England). The smear was sun-dried for 30 minutes and fixed to the slide by passing it through a Benson burner flame. It was stained with ammonium oxalate crystal violet solution for 60 seconds and rinsed with distilled water. The smear was flooded with Lugol's iodine solution for 30 seconds and washed with distilled water, and then decolorized with 70% alcohol and rinsed with distilled water. The smear was counterstained with a safranin solution for 1 minute, rinsed again with water, and blotted with a filter paper. The smear was observed under the microscope in oil immersion using × 100 objective lens [21].

#### 2.5.3. Biochemical Reaction

The pure isolate of DO5 was taken through a series of biochemical tests as indicated in Table 1.

#### 2.5.4. Sugar Fermentation of Isolate DO5

One percent (1%) sterile peptone water of lactose, glucose, galactose, sucrose, mannitol, glycerol, fructose, xylose, starch, pectin, L-sorbose, L-arabinose, raffinose, D-L-alanine, D (+) mannose, chitin, dextrin, and inositol with three drops of 0.01% phenol red. The media were inoculated with a 100 *μ*L culture of isolate DO5 at 37°C for 72 h to observe for acid and gas production [21].

### 2.6. Cultivation of Isolate DO5 on Selective Media

Twenty-four-hour culture of isolate DO5 was streaked on sterile plates of cetrimide agar, bismuth sulfite Agar, MacConkey agar, and mannitol salt agar. The plates were incubated for 24 h at 37°C for growth.

### 2.7. Identification of Isolate DO5 by API and MALDI-TOF Analyses

#### 2.7.1. Analytical Profile Index

Identification of isolate DO5 was done through the analytical profile index (API 20 NE) where six pure colonies of the isolate were picked and suspended in 2 mL of 0.85% NaCl solution. The suspension was adjusted to the turbidity of the 0.5 McFarland standard prior to mixing with AUX-medium. The tube section from nitrate (NO_3_) to p-nitrophenyl-*β*-D-galactopyranoside (PNPG) was filled with the saline solution and 200 *μ*L of isolate DO5 culture. The tube and cupule from glucose (GLU) to phenyl-acetate (PAC) were also filled with AUX-suspension. Sterile mineral oil was added to glucose (GLU), arginine (ADH), and urea (URE) cupules after inoculation to create an anaerobic system and the strip was grown for 18 h at 28°C. The results were read and compared to the API standardized interpretation database.

#### 2.7.2. MALDI-TOF Analysis

The standard ethanol-formic acid (EFA) method was used to prepare isolate DO5 for the matrix-assisted laser desorption ionization-time of flight mass spectrometry (MALDI-TOF). Following the protocol for standard ethanol-formic acid (EFA), isolate DO5 was grown on brain heart infusion agar for 24 h at 30°C. About fifteen (15) pure colonies were picked with sterile disposable plastic inoculating loop into 300 *μ*L sterile distilled water in 2 mL Eppendorf tubes and vortexed. Nine hundred microliters (900 *μ*L) of 96% ethanol were added and thoroughly mixed. The uniform mixture was centrifuged at a speed of 13,000 r.p.m. (revolutions per minute) for 2 minutes and the supernatant was separated from the microbial pellet by pipetting. The pellets were dried at 25°C for 2 minutes and suspended in a 150 *μ*L mixture of 50% acetonitrile and 1% aqueous trifluoroacetic acid in a 2 mL Eppendorf tube containing 20 mg of acid-washed glass beads. The cells were vortexed and centrifuged again for 2 minutes to obtain the extract. One microliter (1 *μ*L) of the extract was sported on MALDI-TOF steel target plate in replicates, dried at 25°C, and then covered with 1 *μ*L of matrix solution (10 mg cyano-4-hydroxycinnamic acid in 1 mL sterile distilled water, 2.5% trifluoroacetic acid, 50% acetonitrile) for co-crystallization of the DNA. The DNA was analyzed with a MALDI-TOF MS spectrometer and the results were interpreted using Bruker Maldi Biotyper Flex control software [24, 25].

### 2.8. Determination of the Effect of Incubation Period on Antimicrobial Activity of DO5 Metabolites

The incubation period for highest metabolites production and maximum activity of DO5 was determined by fermenting the isolate in 360 mL of nutrient broth in a shaking incubator at 37°C for 18 days. After every 24 h intervals, 20 mL aliquots of the culture were pipetted into falcon tubes of 45 mL and centrifuged at 700 r.p.m. for 15 minutes. The cell-free supernatant culture was analyzed for antimicrobial activity against *Klebsiella pneumoniae* using the agar well diffusion method as described in the primary screening of isolated microorganisms for antimicrobial activity protocol above.

### 2.9. Extraction of Metabolites from Isolate DO5

1400 mL of isolate DO5 24 h broth culture was grown in 20 L sterile glucose fermentation medium for 11 days at 37°C. The culture suspension was centrifuged at 700 rpm for 15 minutes and the supernatant was transferred into fresh test tubes. The DO5 supernatant culture was extracted for metabolites with ethyl acetate in a ratio of 1 : 1 using a separating funnel. The ethyl acetate layer was collected into a beaker and recovered using a rotary evaporator (Jos Hansen and Soehne Gmbh R-210, Hamburg, Germany) and the recovered extract was transferred into a preweighed evaporating dish. The extract was dried in a hot air oven at 40°C to a consistent weight and stored at 4°C [26, 27].

### 2.10. Antimicrobial Screening of DO5 Extract Using Agar Well Diffusion and Broth Dilution Methods

#### 2.10.1. Agar Well Diffusion Method

The extract of isolate DO5 was assessed for antimicrobial activity against the test organisms. In the agar well diffusion method, two concentrations (50 mg/mL and 100 mg/mL) of the extract were prepared and tested against *S*. *aureus*, *S*. *pyogenes*, *S*. *typhi*, *K*. *pneumoniae*, *P. mirabilis, V. cholera, E*. *faecalis*, *E*. *coli*, and *C. albicans* in triplicate determination. Ciprofloxacin (12.5 *μ*g/*μ*L) and clotrimazole (25 *μ*g/*μ*L) were used as positive controls [19].

#### 2.10.2. Broth Dilution Method

DO5 extract was analyzed with a broth dilution method to determine the minimum inhibitory (MIC) and minimum bactericidal concentrations (MBC) of the extract in 96 – well microtiter plates. Two-fold dilutions of 100 mg/mL to 0.78 mg/mL concentrations were prepared from the extract. One hundred microliters (100 *μ*L) of the double strength nutrient broth and 80 *μ*L of the extract were dispensed into 96-well plates followed by the addition of 20 *μ*L of the test microbes' suspension (1.0 × 10^−5^ CFU/mL) to make a final volume of 200 *μ*L. All the plates were incubated for 24 h at 37°C after which 20 *μ*L of 1.25 mg/mL of tetrazolium salt (3-(4,5-dimethylthiazole-2yl-2,5-diphenyltetrazolium bromide) solution was added and incubated at 37°C for 30 min. Wells that developed a purple color after addition of the MTT indicated growth of test microbes and those that retained the yellow color indicated growth inhibition of the test microbes [19]. One hundred (100) microliters of cultures were pipetted from wells that showed inhibitory activity and subcultured into sterilized nutrient broths by incubation at 37°C for 24 h. The least concentration did not show microbial growth after the incubation period was considered the minimum bactericidal concentration.

### 2.11. Chromatographic Analyses of the Isolate DO5 Extract

#### 2.11.1. Thin-Layer Chromatography

DO5 extract of 10 mg/mL in methanol was profiled along the horizontal line drawn on a TLC plate of dimensions 2 × 8 cm. The plates were developed in a petroleum ether-chloroform-methanol (80 : 10 : 10) solvent system and viewed under a UV lamp at a wavelength of 254 and 365 nm. The spots were circled with pencil and plates were sprayed with 1 mL anisaldehyde solution, air-dried, and heated at 100°C for 5 minutes in an oven for visibility of spots. Distance travelled by the spots and the solvent front were measured in cm with the aid of a meter ruler and used to calculate retardation factor (*R*_*f*_) values.

#### 2.11.2. Column Chromatography

About 2.74 g DO5 extract was dissolved in 5 mL ethyl acetate and mixed with 6.0 g silica gel to form a slurry. The dried slurry was chromatographed over a silica gel column (70–230 mesh–Merck) and eluted with 100% petroleum ether of increasing solvent polarity from ethyl acetate to methanol in a ratio of 90 : 10, 80 : 20, 70 : 30, 60 : 40, 50 : 50, 40 : 60, 30 : 70, 20 : 80, and 10 : 90. Forty-four (44) fractions were collected in 10 mL beakers, air-dried, and bulked into eight subfractions according to their TLC profile [28].

#### 2.11.3. Gas Column Chromatography-Mass Spectrometry

One microliter of the bioactive fraction 50 *μ*g/*μ*L concentration was analyzed on PerkinElmer Clarus 580 and (Clarus SQ 8 S) GC/MS using Elite-5 MS 5% diphenyl/95% dimethylpolysiloxane column dimensions of 30 × 0.25 *μ*m ID × 0.25 *μ*m DF. The temperatures for injector and ion source were maintained at 250°C and 150°C, respectively. Detection of GC-MS was operated in electron impact mode with ionization energy of 70 electronvolts (eV) using helium gas of 99.99% purity as a carrier gas at a stable flow rate of 1 mL/min. The mass spectrum of the bioactive fractions was read at 70 eV, scanned within 0.5 seconds, and fragmented from 45 to 450 Da following their mass to charge ratio. The separated ions were detected by the Turbo-Mass detector and amplified signals of the spectra to Turbo-Mass version-6.1.0 software for recording the chromatogram. The solvent was started at zero minute and delayed for 2 minutes before the GC-MS was run for 47 minutes to initialize the system. The mass spectra of the bioactive fractions determined were interpreted and compared to the GC-MS spectrum database of the National Institute Standard and Technology (NIST).

### 2.12. Infrared Spectroscopic Analysis of DO5 Bioactive Fraction

An attenuated total reflectance infrared spectrophotometer was used to determine the number of functional groups present in the DO5 bioactive fraction (BF2124). Ten milligrams (10 mg) of dried fractions were mounted directly on the KBr disc of the PerkinElmer 200 UATR (FT-IR C9413) spectrophotometer [29] and scanned through the IR region between the ranges of 400 to 4000 cm^−1^ at a resolution of 4 cm^−1^.

### 2.13. Statistical Analysis

Data were analyzed with GraphPad Prism version 8.0 for Windows (GraphPad Software Inc., San Diego, CA, USA). One-way ANOVA followed by Dunnett's *post hoc* test was conducted.

## 3. Results

### 3.1. Isolation and Antimicrobial Screening

A total of 48 isolates suspected of having the capacity to produce antimicrobial metabolites were isolated from 36 samples collected, out of which only 10 showed zones of growth inhibition ranging from 12.50 ± 0.707 to 21.50 ± 0.701 mm against at least one of the test organisms used (Table 2). One of the isolates inhibited *Proteus mirabilis;* five inhibited *Klebsiella pneumoniae;* four inhibited *E. coli* and *Candida albicans;* three inhibited *Pseudomonas aeruginosa;* two inhibited *Salmonella typhi*, *Enterococcus faecalis,* and *Staphylococcus aureus;* while one inhibited *Streptococcus pyogenes* and *Vibrio cholera* (Table 2). In all, isolate DO5 exhibited a broad spectrum of activity against Gram-positive and Gram-negative bacteria including fungi and was selected for further studies.

### 3.2. Morphological and Biochemical Characterization of Isolate DO5

Gram's staining and microscopic examination presented isolate DO5 as Gram-negative rod-like bacteria that reacted with 3% KOH solution to produce a stringy or mucoid reaction. The isolate grew in 1 to 3% NaCl solution but not in 5 to 7% w/v. It also showed growth from pH 7 to 9 but not at pH 4 to 6. The isolate grew on MacConkey agar, bismuth sulfite agar, cetrimide agar, and Sabouraud agar but not on mannitol salt agar. The isolate was a motile bacterium which did not produce indole, H_2_S gas, and VP but produced acid, oxidase, catalase, methyl red, and citrate (Table 3).

### 3.3. API 20 NE and MALDI-TOF Identification of Isolate DO5

All values corresponding to the positive reactions in each group were added to generate a seven-digit number (7). Based on the numbered profile of API 20 NE analytical index book, isolate DO5 was identified to be *P. aeruginosa* (ID ≥ 99.9% and *T* ≥ 0.9). The isolate identification was confirmed to the species level by matrix-assisted laser.

### 3.4. Effect of Carbon Sources, Potassium Nitrate, Calcium Carbonate, and Glucose Concentrations on Antimicrobial Activity of Isolate DO5

The isolate produced metabolites in maltose, galactose, sucrose, glucose, fructose, mannitol, lactose, and glycerol media which exhibited activity against *S. aureus, E. coli*, *K. pneumoniae*, and *C. albicans*. Metabolites in the xylose medium produced no activity against the organisms (Figure S1). The bioactive metabolites produced in the potassium nitrate medium showed growth inhibitory action against the test organisms at all concentrations. The highest activity was observed at concentrations between 0.1 and 0.5% (Figure S2). The metabolites produced in the CaCO_3_ medium also exhibited activity against all test organisms at concentrations of 0.1–1.0% (Figure S3). Bioactive metabolites produced by DO5 in the glucose medium showed activity against the test organisms at concentrations of 0.1–1.0% with *Candida albicans* showing the least activity (Figure S4).

### 3.5. Incubation Period and Fermentation

**Figure 1:**
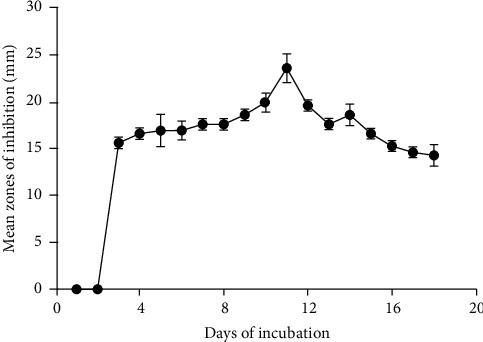
Incubation time effect on antimicrobial activity of isolate DO5 against *K. pneumoniae*.

Isolate DO5 produced antimicrobial metabolites for maximum activity from day 3 with the highest zone of growth inhibition of 23.67 mm occurring on day 11. Whereas, the least activity of 14.33 mm was recorded on day 18 against *K. pneumoniae* (Figure 1). Aliquots of isolate DO5 fermented supernatant which were taken every 24 h produced inhibitory activity of 17.00 ± 1.41, 17.59 ± 2.99, 17.50 ± 0.71 and 18.00 ± 00 mm against *S. aureus, C. albicans, K. pneumoniae,* and *E. coli,* respectively, in agar well determination (Table 4).

### 3.6. Extraction and Antimicrobial Activity of the Crude DO5 Extract

The dark brown crude extract (3.59 g) obtained after the fermentation process exhibited activity against *S. aureus, E. faecalis, E. coli*, *K. pneumoniae*, *V. cholerae*, S*. typhi*, *P. aeruginosa*, *S. pyogenes*, *C. albicans,* and *P. mirabilis* with zones of inhibition ranging between 11.67 ± 0.23 and 21.50 ± 0.71 mm (Table 5). The MIC and MBC ranged from 3.13–25.0 mg/mL and 6.25–50.0 mg/mL, respectively, against test organisms. Ciprofloxacin inhibited MIC of 1.56 *μ*g/mL to 12.5 *μ*g/*μ*L and MBC of 3.12 *μ*g/*μ*L to 25.0 *μ*g/*μ*L. Ketoconazole exhibited MIC and MBC of 25.0 *μ*g/*μ*L and 50.0 *μ*g/*μ*L against *C. albicans*, respectively (Table 6).

### 3.7. TLC Analysis and Fractionated Bioactive Fractions from DO5 Extract

TLC of DO5 crude extract revealed five 5 components under UV light at 254 nm and 3 components at 365 nm with the *R*_*f*_ values of the spots (Table S1). Forty-four (44) bioactive fractions were collected from DO5 extract after fractionation and bulked into eight subfractions of BF2124, BF2529, BF3135, BF5160, BF6166, BF6770, BF7175, and BF7679. All subfractions exhibited antimicrobial activity against *S. aureus* with subfraction BF2124 recording the highest antimicrobial activity (Table 7).

### 3.8. Infrared (IR) and Gas Chromatography-Mass Spectroscopy Analysis

IR analysis recorded 1039.77, 1072.78, 1122.72, 1272.84, 1378.02, 1464.55, 1601.47, 1727.54, 2853.41, and 2922.87 cm as peaks for functional groups in the BF2124 bioactive fraction. The functional groups revealed at the above peaks were ***υ*(C-O)** vibrational frequency of carbon-oxygen; *υ ***(C-H)** vibrational frequency of the saturated carbon-hydrogen bond; *υ ***(C-C)** vibrational frequency of the carbon-carbon saturated bond; *υ ***(C=C)** vibrational frequency of the carbon-carbon unsaturated bond; *υ ***(C=O)** vibrational frequency of the carbon-oxygen double bond (Table 8). Nine (9) compounds were detected in the DO5 bioactive fraction (BF2124) by GC/MS analysis at retention time (RT) of 16.86 mins, 23.05 mins, 21.18 mins and 24.35 mins, of 16.864 mins, 19.125 mins, 21.181 mins, 23.047 mins, 24.352 mins, 25.568 mins, 25.603 mins, 26.053 mins, and 26.528 mins. Compounds identified were E-15-heptadecenal, 1-docosene, 3-eicosene, 1-eicosanol, 1-nonadecene, 1-hexadecanol, pyrrolizidine (pyrrolo (1,2-a) pyrazine-1,4-dione, 1-hexadecene, and 1-heptacosanol (Table 9).

## 4. Discussion

The growing negative impact of antimicrobial resistance has necessitated the search for newer and effective microbial agents from varying sources including microorganisms. River bodies and landfill sites have been reported to be major sites for obtaining antibiotic-producing bacteria. This study therefore reports the isolation of antibiotic-producing organisms from soil and water samples taken from the Dompoase landfill site, KNUST Physic Garden, Kosiko River, and Ada Foah seashore. Ten isolates were identified to be antibiotic-producing organisms. This could be due to the ecological role such as defensive mechanism to maintain their niche, or invasion of an established microbial community [30]. It has been reported that live and heat-killed cells of *S. aureus* induced the production of secondary metabolites from marine microbial isolates [31]. About 119 microorganisms were screened and only 27 isolates were found active against one test organism [18]. Fifty-seven (57) bacteria isolates were screened and only 2 showed activity [32]. In another investigation, 23 bacteria isolates tested for antimicrobial activity and only 7 isolates were active [33]. In this current study, ten (10) out of 48 isolates exhibited antimicrobial activity against the test microorganisms. The supernatant culture of isolate DO5 exhibited activity against the test organisms which indicated that metabolites are not produced only in the existence of competitions but can be produced in a medium that contains appropriate nutrients and at favorable temperature [32, 34]. The activity of metabolites produced by isolate DO5 started on the 3^rd^ day of incubation and increased daily to the 11^th^ day. Many factors control the production of secondary metabolites and the principal factor among them is the formulation ingredients of the medium [32, 34]. Differences that may occur in the fermentation media can result in changes in the size of inhibition zones produced by the metabolites. Isolate DO5 was found to produce high antimicrobial activity against test microbes in the fermentation media enriched with maltose, galactose, sucrose, glucose, fructose lactose, mannitol, and glycerol. From this study, isolate DO5 was morphologically reported as Gram-negative, rod-shaped, motile, and oxidase-positive and grew on all the selective media except mannitol salt agar because it does not tolerate high salt concentration. DO5 was indole-negative but catalase-positive. *Pseudomonas aeruginosa,* AZ-SH-B8, was identified using API 20 NE kit during the production of antibiotic sparsomycin and recorded similar results such as nitrate reduction activity, negative indole production, and urease and oxidase production [35].

Isolate DO5 was identified to the species level by MALDI-TOF mass spectrometry as *Pseudomonas aeruginosa* score value was greater than or equal to 2.35 [36, 37]. The score value represents the identification of isolate DO5 to the species level. The extract of isolate DO5 had activity on all the test organisms. The MIC recorded ranged between 3.12 and 25 mg/mL whereas the MBC between 6.25 and 50 mg/mL. The TLC analysis revealed eight spots of bioactive compounds from the extract with different retardation factors. An infrared spectrum of the DO5 bioactive fraction showed some characteristic peaks due to the presence of functional groups such as C-O, C=O, C-H, and C=C. From the infrared studies on purification and physiochemical characterization of pyomelanin pigment produced from local *Pseudomonas aeruginosa* isolates showed presence of similar (C=O, C-H and C=C) functional groups [38]. The absorption peaks of the pigment resembled peaks of the bioactive DO5 fraction but pyomelanin was not found in the DO5 fraction which may be due to differences in geographical locations. Gas chromatography-mass spectrophotometry analysis conducted by Altaee et al. [39] identified oxime-, methoxy-phenyl, Edulan II, methyl-4-nitromethyl-4-qpiperidinol, acetamide, N-methyl-N-4-2-fluoromethyl-1-pyrrolidyl-2-buty, oxaspiro 4,4 nonane-4-one, 2-isopropyl, octahydrochromen-2-one, 3,7-diazabicyclo 3.3.1 nonane, 9,9-dimethyl, N-3-N-aziridyl propylidene tetrahydrofurfurylamine, benzenemethanol-aminopropoxy-3-methyl, dithiocarbamate, dl-Allo-cystathionine, deoxyspergualin, and dl-2,6-diaminoheptanedioic acid from *Pseudomonas aeruginosa* isolated from urinary tract infection patients. None of these compounds were found in the DO5 bioactive fraction which may be due to differences in their geographical locations. Results from the current GC-MS analysis of the DO5 fraction suggested bioactive compounds such as E-15-heptadecenal, 1-docosene, 3-eicosene, 1-eicosanol, 1-nonadecene, 1-hexadecanol, pyrrolizidine (pyrrolo) 1,2-a pyrazine-1,4-dione, 1-hexadecene, and 1-heptacosanol. The presence of these compounds could be responsible for the antimicrobial activity of DO5.

## 5. Conclusion

The ethyl acetate extract of isolate DO5, identified to be a strain of *Pseudomonas aeruginosa*, exhibited promising antimicrobial activity against *Staphylococcus aureus* (ATCC 25923), *Enterococcus faecalis* (ATCC 29212), *Escherichia coli* (ATCC 25922), *Pseudomonas aeruginosa* (ATCC 4853) *Streptococcus pyogenes, Klebsiella pneumoniae, Proteus mirabilis, Vibrio cholera*, *Salmonella typhi*, and *Candida albicans*. The fractions of isolate DO5 extract showed broad-spectrum activity against test organisms. The ethyl acetate extract of isolate DO5 is a potential lead for antimicrobial compounds.

## Figures and Tables

**Table 1:** Biochemical tests performed.

Biochemical test	Inference	Reference
Catalase production	Evolution of oxygen gas which indicated the presence of catalase enzyme which converts H_2_O_2_ to H_2_O and O_2_	[22]
Oxidase production	A colour change from purple to deep blue indicated the presence of oxidase enzyme	[22]
Hydrogen sulphide (H_2_S) production	Suspended lead acetate paper turns black	[22]
Potassium hydroxide reaction	Thickening slurry which is a characteristic of Gram-Negative bacteria	[22]
Indole production	Red ring colouration on the surface of the suspension	[22]
Methyl red Voges-Proskauer (MRVP)	Red colour in the medium was a positive reaction for MR Strong red colouration indicated a positive VP test	[23]
Nitrate reduction	Red colouration in media	[22]
Citrate utilisation	Colour change from light blue to green	[23]
Urease production	Production of a pink colour	[23]
Motility	Diffused hazy growth extending out from the line of inoculation	[23]
Starch hydrolysis	Occurrence of a clear zone around the colony	[21]

**Table 2:** Antimicrobial activity of isolate DO5 using the agar well diffusion method.

Isolates	Test microorganisms/Mean zones of inhibition (mm)									
	ST	CA	EF	EC	PA	KP	SA	SP	PM	VC
DO1	—	—	—	—	15.50 ± 0.707	—	—	—	17.50 ± 0.707	—
DO2	—	16.50 ± 0.707	—	—	—	—	—	—	13.50 ± 0.707	—
DO5	15.0 ± 1.414	17.50 ± 0.707	19.50 ± 0.707	21.50 ± 0.707	16.0 ± 1.414	19.50 ± 0.707	19.50 ± 1.414	16.50 ± 1.414	15.50 ± 1.414	15.50 ± 0.707
DO6	—	—	—	—	15.50 ± 0.707	—	—	—	17.50 ± 0.707	—
DO7	—	16.50 ± 0.707	—	—	—	—	—	—	13.50 ± 0.707	—
ACT301	—	—	—	18.50 ± 2.121	—	—	—	—	—	—
ACT302	—	—	—	—	—	17.50 ± 2.12	—	—	19.50 ± 1.414	—
ACT303	—	—	16.50 ± 0.707	—	—	15.0 ± 0.00	—	—	12.50 ± 0.707	—
ACT304	—	16.50 ± 0.707	—	15.50 ± 0.707	—	19.50 ± 1.414	14.50 ± 0.707	—	—	—
ACT307	16 ± 2.828	—	—	13.50 ± 0.707	—	15.50 ± 2.12	—	—	—	—

ST-*Salmonella typhi*; CA-*Candida albicans*; EF-*Enterococcus faecalis*; EC-*Escherichia coli*; PA-*Pseudomonas aeruginosa*; KP-*Klebsiella pneumonia;* SA-*Staphylococcus aureus*; ST-*Streptococcus pyogenes*; PM-*Proteus mirabilis;* VC-*Vibrio cholerae*.

**Table 3:** Morphological and biochemical properties of isolate DO5.

Characteristics	Results
Colour of colonies	Gray
Gram reaction	Negative
Motility	+
Cell shape	Rod
Oxidase test	+
Catalase test	+
Starch hydrolysis	+
*Biochemical characteristics*	
H_2_S production	−
Nitrate reduction	+
Urease test	+
Citrate test	+
Indole test	−
Methyl red test	+
Voges-Proskauer test	−
Reaction with 3% KOH	+
*Acid Production*	
Glucose	+
Maltose	+
Galactose	+
Sucrose	+
Mannitol	+
Glycerol	+
Lactose	+
Fructose	+
Xylose	+
Gelatin	+
Starch	+
*Acid production*	
Pectin	−
L-sorbose	−
L-arabinose	−
Raffinose	−
DL-alanine	+
D (+) mannose	−
Chitin	+
Dextrin	+
Insulin	−
Inositol	+
NaCl (1–3%)	+
NaCl (5–7%)	−
pH (4–6)	−
pH (7–9)	+
*Growth on selective media*	
MacConkey agar	+ (Brown)
Mannitol agar	−
Sabouraud dextrose agar	+ (yellow)
Bismuth sulphite agar	+ (green)
Cetrimide agar	+ (green)

(+) = Positive activity, (−) = Negative activity.

**Table 4:** Antimicrobial activity of supernatant of isolate DO5.

Isolate	Mean zones of inhibition (mm)^*∗*^			
	*S. aureus*	*E. coli*	*K. pneumoniae*	*C. albicans*
DO5	17.00 ± 1.41	18.00 ± 00	17.50 ± 0.71	17.59 ± 2.99

^*∗*^ *n* = 3.

**Table 5:** Antimicrobial activity of DO5 extract.

Test organisms	Mean zones of inhibition (mm)		Ciprofloxacin	Ketoconazole
	100 mg/mL	50 mg/mL	12.5 *μ*g/mL	50 *μ*g/mL
*S. aureus*	17.00 ± 1.41^a^	16.50 ± 1.41a	19.50 ± 0.707 b	ND
*S. pyogenes*	18.50 ± 0.71^a^	16.50 ± 0.71^b^	16.00 ± 0.707^b^	ND
*E. coli*	19.50 ± 2.12^a^	15.50 ± 2.12^b^	18.00 ± 1.414^a^	ND
*E. faecalis*	17.00 ± 1.42^a^	15.00 ± 1.42^b^	20.50 ± 0.707^c^	ND
*P. aeruginosa*	13.83 ± 0.76^a^	11.67 ± 0.23^b^	16.50 ± 0.707^c^	ND
*S. typhi*	15.63 ± 0.71^a^	14.06 ± 1.21^a^	14.50 ± 0.707^a^	ND
*P. mirabilis*	15.50 ± 0.71^a^	14.03 ± 0.55^a^	19.50 ± 0.707^b^	ND
*K. pneumonia*	21.00 ± 1.42^a^	16.50 ± 1.42^b^	22.50 ± 0.707^a^	ND
*C. albicans*	17.50 ± 0.71^a^	16.50 ± 0.71^a^	ND	16.50 ± 0.71^a^
*V. cholerae*	16..23 ± 0.25^a^	12.83 ± 0.76^b^	15.00 ± 1.414^a^	ND

ND─not determined, *n* = 3. Same alphabets indicate not significant statistical difference (*P* > 0.05), whereas different alphabets on the same row indicate significant statistical difference (*P* < 0.05).

**Table 6:** MIC and MBC of DO5 extract, ciprofloxacin, and ketoconazole.

Test organisms	DO5 extract (mg/mL)		Ciprofloxacin (*μ*g/mL)		Ketoconazole (*μ*g/mL)	
	MIC	MBC	MIC	MBC	MIC	MBC
*S. aureus*	3.12	6.25	1.56	3.12	ND	ND
*E. coli*	6.25	12.5	12.5	25.0	ND	ND
*E. faecalis*	3.12	6.25	6.25	12.5	ND	ND
*K. pneumoniae*	6.25	12.5	6.25	12.5	ND	ND
*P. mirabilis*	12.5	25.0	3.12	6.25	ND	ND
*V. cholerae*	6.25	12.5	3.12	6.25	ND	ND
*P. aeruginosa*	25.0	50 .0	12.5	25.0	ND	ND
*S. pyogenes*	25.0	50 .0	12.5	25.0	ND	ND
*S. typhi*	6.25	12.5	3.12	6.25	ND	ND
*C. albicans*	25.0	50.0	ND	ND	25.0	50.0

ND-not determined, *n* = 3.

**Table 7:** Antimicrobial activity of DO5 bioactive subfractions against *S. aureus*.

Number	Bioactive subfractions	Mean zones of inhibition (mm)
1	BF2124	25.50 ± 0.71
2	BF2529	18.50 ± 0.71
3	BF3135	15.00 ± 0.00
4	BF5160	14.50 ± 0.71
5	BF6166	21.50 ± 0.71
6	BF6770	22.50 ± 3.54
7	BF7175	20.50 ± 0.71
8	BF7679	19.00 ± 1.41

BF-Bioactive fraction; *n* = 3.

**Table 8:** IR spectrum of functional groups present in DO5 bioactive fractions.

Peak values (cm^−1^)	Intensity	Functional groups	Types of vibration
**1039.77**	Strong	**(C–O)** (primary alcohol)	Stretching
**1072.78**	Strong	**(C–O)** (primary alcohol)	Stretching
**1122.72**	Strong	**(C–O)** (secondary alcohol)	Stretching
**1272.84**	Strong	**(C–O)** (aromatic ester)	Stretching
**1378.02**	Medium	**(C–H)** (alkane)	Bending
**1464.55**	Medium	**(C–C)** (aromatics)	Stretching (in-ring)
**1601.47**	Medium	**(C=C)** (conjugate alkene)	Stretching
**1727.54**	Strong	**(C=O)** (*α*, *β*, unsaturated ester/formate)	Stretching
**2853.41**	Medium	**(C–H)** (alkane)	Stretching
**2922.87**	Medium	**(C–H)** (alkane)	Stretching

**Table 9:** Compounds identified in DO5 subfraction (BF2124) by GC-MS analysis and a review of their documented antimicrobial activities.

Peak no	Retention time (min)	Compound name	Molecular formula	Molecular weight	Area (%)	Normal (%)	Compound nature	Biological activity	References
1	16.864	E-15-heptadecenal	C_17_H_32_O	252.44	0.386	2.70	Long-chain alkene	Antimicrobial and antioxidant	[27, 28]
2	19.125	3-Eicosene	C_20_H_40_	280.53	0.482	3.38	Long-chain fatty acid	Antimicrobial	[29]
3	21.181	1-Nonadecene	C_19_H_38_	266.51	0.509	3.56	Alkene (long-chain fatty acid)	Antimicrobial and antifungal	[30, 31]
4	23.047	1-Eicosanol	C_20_H_42_O	298.00	0.346	2.42	Alcohol	Antimicrobial	[32]
5	24.352	1-Docosene	C_22_H_44_	308.00	0.457	3.20	Alkene	Antimicrobial	[30, 31]
6	25.568	1-Hexadecanol	C_16_H_34_O	242.00	2.191	15.34	Alcohol	Antimicrobial	[33, 34]
7	25.603	Pyrrolo (1,4-dione) hexahydro-3-2-methylpropyl 1, 2apyrazine	C_11_H_18_N_2_O_2_	210.00	1.050	7.35	Alkaloid pyroolizidine	Antimicrobial and anti-inflammatory	[35]
8	26.053	1-Heptacosanol	C_27_H_56_O	396.73	0.347	2.43	Long fatty alcohol	Antifungal, antimicrobial, antiviral, and antioxidant	[36]
9	26.528	1-Hexadecene	C_16_H_32_	224.42	2.319	16.24	Alkene	Antifungal, antimicrobial, and antioxidant	[37, 38]

## Data Availability

The datasets used and/or analyzed during the current study are included within the article. Further clarification can be obtained from the corresponding author.

## Supplementary Materials

Supplementary Materials In the supplementary data, the methodology for the determination of the effect of different sugars (glucose, galactose, xylose, sucrose, mannitol, lactose, fructose, glycerol, and maltose) and varying concentrations of glucose, potassium nitrate, and calcium carbonate on the antimicrobial activity of isolate DO5 is described. The results for the experiments described above as well as the TLC analysis (showing spots and their respective Rf values) of crude extract of isolate DO5 are available in the supplementary data.
