# Supplementary material for: Antimicrobial Potential of Extract from a Pseudomonas aeruginosa Isolate
Source: Scientifica (Cairo). 2022 Jun 29;2022:4230397. doi: 10.1155/2022/4230397 (PMC9259217; doi:10.1155/2022/4230397)
Supplement: Supplementary Materials — In the supplementary data, the methodology for the determination of the effect of different sugars (glucose, galactose, xylose, sucrose, mannitol, lactose, fructose, glycerol, and maltose) and varying concentrations of glucose, potassium nitrate, and calcium carbonate on the antimicrobial activity of isolate DO5 is described. The results for the experiments described above as well as the TLC analysis (showing spots and their respective Rf values) of crude extract of isolate DO5 are available in the supplementary data. [file 4230397.f1.docx]

**SUPPLEMENTARY DATA**

**Methodology**

**Determination of the effect of growth factors on antimicrobial activity of DO5 metabolites**

***Carbon sources***

One gram (1.0 g) of glucose, galactose, xylose, sucrose, mannitol, lactose, fructose, glycerol and maltose were separately added to test tubes containing 10 mL fermentation medium and sterilized (composition of glucose 0.2 g, Sodium nitrate 0.2 g, Dipotassium hydrogen phosphate 0.11 g, Magnesium sulphate 0.5 g, Sodium chloride 0. 25 g, Calcium carbonate 0.1 g, Distilled water 1L and pH of 7.2). One millilitre of organism DO5 suspension was inoculated into each of the media aseptically and incubated for 11 days at 37ºC. After incubation, the cultures were centrifuged and the supernatant was assayed against *S. aureus, E. coli, K. pneumoniae* and *C. albicans* using the agar well diffusion method for antimicrobial activity.

***Glucose concentrations***

Ten test tubes containing 10 mL of sterile fermentation medium were separately supplemented with different concentrations (0.1 - 1.0%) of glucose and then inoculated with 1mL of isolate DO5. The tubes were cultured in an incubator at 37ºC for 11 days after which they were centrifuged. The supernatants were tested using the agar well diffusion method in triplicates for activity against *S. aureus, E. coli, K. pneumoniae* and *C. albicans*.

***Nitrogen source (potassium nitrate) concentrations***

Test tubes containing 10 mL of sterile fermentation medium were separately supplemented with different concentrations (0.1- 1.0%) of potassium nitrate and then inoculated with 1 mL of isolate DO5. The setups were grown using an incubator at 37ºC for 11 days after which they were centrifuged. Antimicrobial activity of the supernatants was tested in triplicates against *S. aureus, E. coli, K. pneumoniae* and *C. albicans* using the agar well diffusion method.

***Calcium carbonate (CaCO_3_) concentrations***

Ten test tubes containing 10 mL of sterile fermentation medium were separately supplemented with different concentrations (0.1- 1.0%) of CaCO_3_ and then inoculated with 1 mL of isolate DO5. The setups were cultured in an incubator for 11 days at 37ºC after which they were centrifuged. The activity of the supernatants was assayed against *S. aureus, E. coli, K. pneumoniae* and *C. albicans* by the agar well diffusion method.

**Supplementary results**

**Effect of carbon sources, potassium nitrate, calcium carbonate and glucose concentrations on antimicrobial activity of isolate DO5**

***Carbon sources***

The isolate produced metabolites in maltose, galactose, sucrose, glucose, fructose, mannitol, lactose and glycerol media which exhibited activity against *S. aureus,* *E. coli*, *K. pneumoniae* and *C. albicans*. Metabolites in xylose medium produced no activity against the organisms (Figure S1).

Figure S1: Effect of carbon sources on antimicrobial activity of isolate DO5

***Potassium nitrate concentration***

The bioactive metabolites produced in the potassium nitrate medium showed growth inhibitory action against the test organisms at all concentrations. The highest activity was observed at concentrations between 0.1 and 0.5% (Figure S2).

Figure S2: Effect of various concentrations of potassium nitrate in the medium on bioactivity of DO5 metabolite.

***Calcium carbonate concentration***

The metabolites produced in the CaCO_3_ medium also exhibited activity against all test organisms at concentrations of 0.1 – 1.0% (Figure S3).

Figure S3: Effect of various concentrations of calcium carbonate in the medium on bioactivity of DO5 metabolite.

***Glucose concentration***

Bioactive metabolites produced by DO5 in the glucose medium showed activity against the test organisms at concentrations of 0.1 - 1.0% with *Candida albicans* showing the least activity (Figure S4).

 Figure S4: Effect of various concentrations of glucose in the medium on bioactivity of DO5 metabolite

**TLC analysis and fractionated bioactive fractions from DO5 extract**

TLC of DO5 crude extract revealed five 5 components under UV light at 254 nm and 3 components at 365 nm with the R_f_ values of the spots (Table S1).

Table S1. TLC Spots with their respective wavelength and retardation factors

| Spot | Wavelength (nm) | Retardation factor (Rf) |
| --- | --- | --- |
| 1 | 365 | 0.053 |
| 2 | 254 | 0.147 |
| 3 | 254 | 0.221 |
| 4 | 254 | 0.305 |
| 5 | 254 | 0.536 |
| 6 | 254 | 0.642 |
| 7 | 365 | 0.768 |
| 8 | 365 | 0.894 |
